# High-throughput characterization of chemical-associated embryonic behavioral changes predicts teratogenic outcomes

**DOI:** 10.1007/s00204-015-1554-1

**Published:** 2015-07-01

**Authors:** David M. Reif, Lisa Truong, David Mandrell, Skylar Marvel, Guozhu Zhang, Robert L. Tanguay

**Affiliations:** Bioinformatics Research Center, Department of Biological Sciences, North Carolina State University, Raleigh, NC USA; Sinnhuber Aquatic Research Laboratory, Department of Environmental and Molecular Toxicology, Environmental Health Sciences Center, Oregon State University, 28645 East Highway 34, Corvallis, OR 97333 USA

**Keywords:** Developmental neurotoxicology, Alternative testing, Chemical biology, Behavior, Zebrafish, High-throughput screening, Bioinformatics, Data integration, ToxCast, Bioactivity

## Abstract

New strategies are needed to address the data gap between the bioactivity of chemicals in the environment versus existing hazard information. We address whether a high-throughput screening (HTS) system using a vertebrate organism (embryonic zebrafish) can characterize chemical-elicited behavioral responses at an early, 24 hours post-fertilization (hpf) stage that predict teratogenic consequences at a later developmental stage. The system was used to generate full concentration–response behavioral profiles at 24 hpf across 1060 ToxCast™ chemicals. Detailed, morphological evaluation of all individuals was performed as experimental follow-up at 5 days post-fertilization (dpf). Chemicals eliciting behavioral responses were also mapped against external HTS in vitro results to identify specific molecular targets and neurosignalling pathways. We found that, as an integrative measure of normal development, significant alterations in movement highlighted active chemicals representing several modes of action. These early behavioral responses were predictive for 17 specific developmental abnormalities and mortality measured at 5 dpf, often at lower (i.e., more potent) concentrations than those at which morphological effects were observed. Therefore, this system can provide rapid characterization of chemical-elicited behavioral responses at an early developmental stage that are predictive of observable adverse effects later in life.

**Electronic supplementary material:**

The online version of this article (doi:10.1007/s00204-015-1554-1) contains supplementary material, which is available to authorized users.

## Introduction

There are tens of thousands of manufactured chemicals currently in commerce, each with varying production volumes, use profiles, and potential for environmental release, persistence, and bioaccumulation. Comprehensive data on the human and environmental health hazards of these chemicals remain elusive, ranging from sparse to nonexistent. High-throughput screening (HTS) efforts such as ToxCast™ and Tox21™ were conceived to begin addressing this data need by applying new in vitro assay technologies to speed the pace of chemical testing (Collins et al. 2008; Judson et al. 2010).

While simple in vitro screening assays are useful for probing targeted receptor–ligand interactions, these screens fail to take into account the complexity of the vertebrate nervous system and will miss chemicals that modify nervous system function in novel ways. A phenotype-based screen is needed to identify developmental neuromodulatory compounds in the absence of specific targets (Burns et al. 2013; Selderslaghs et al. 2013). Designing in vivo phenotypic screens in a high-throughput manner would provide the ability to discover complex behavioral phenotypes using whole organisms, where the full gamut of coordinated events leading to nervous system development occurs, including cellular differentiation, proliferation, migration, synapse formation, and apoptosis (Makris et al. 2009; Padilla et al. 2012; Sanes et al. 2005; Truong et al. 2014).

Zebrafish (*Danio rerio*) is a prolific, small, complex organism that shares a highly conserved anatomy and physiology with all vertebrates (Howe et al. 2013). Importantly, the critical processes of zebrafish neurodevelopment are homologous to those in humans (Tropepe and Sive 2003). Early in zebrafish embryogenesis [roughly 19–29 hours post-fertilization (hpf)], spontaneous tail contractions occur as the muscles in this region are innervated (Kimmel et al. 1995). This spontaneous behavior is sensitive to high-intensity light perturbation via photoreceptors in the developing hindbrain and has been designated as the photomotor response (PMR) (Kokel et al. 2013). The normal PMR is sensitive to chemical perturbation and amenable to screening for behavior-modifying compounds (Raftery et al. 2014). In Kokel et al. (2010), the authors developed behavioral barcodes that classified small molecules from six libraries (primarily pharmaceuticals and candidate pharma-like chemical entities) into related clusters according to PMR of pooled embryo samples at a single concentration.

Here, we report HTS results that characterize the diverse ToxCast Phase-I and Phase-II libraries (1060 unique chemicals) using a five-point, log concentration range (from 6.4 nM to 64 µM) for each chemical. These results were generated using single-embryo wells with 32 replicate samples at each concentration, where all individuals are followed up by morphological assessments at 120 hpf (5 dpf). Thus, our assay system allows the characterization of behavioral changes across chemical concentrations, provides increased detection power and reliability of individual endpoint measurements, and quantifies developmental consequences of behavioral aberrations measured early in development. As additional follow-up, we mapped our results to external data to discover associations between behavioral responses and in vitro endpoints targeting specific biological pathways.

## Methods

### Chemicals

The ToxCast Phase-I and Phase-II chemical libraries were provided by the US EPA National Center for Computational Toxicology (NCCT). There were 1060 unique chemicals from various sources. Stock solutions of all the chemicals were provided at a 20-mM concentration in 100 % dimethyl sulfoxide (DMSO) in multiple 96-well plates. More details regarding the chemical library and quality control can be found at http://www.epa.gov/NCCT/toxcast/chemicals.html. Chemical dilutions (1:10) for exposure plates were made using DMSO and standard embryo medium (EM) (Westerfield 2000) to achieve a concentration range of 0.064–640 µM with a DMSO concentration of 6.4 %. All dilution plates were sealed and then stored at −20 °C until used for exposure.

### Experimental design

Adult wild-type zebrafish (Tropical 5D) were raised in the Sinnhuber Aquatic Research Laboratory at Oregon State University, Corvallis, Oregon. Groups of 1000 adult zebrafish were housed in 100 gallon tanks kept at standard laboratory conditions of 28 °C on a 14-h light/10-h dark photoperiod in fish water (reverse osmosis water supplemented with Instant Ocean™, a commercially available salt). Zebrafish were spawned, and embryos were collected and staged according to Kimmel et al. (Kimmel et al. 1995). At 4 hpf, embryos were dechorionated using pronase (63.6 mg/ml, >3.5 U/mg, Sigma-Aldrich: P5147) by a custom automated dechorionator (Mandrell et al. 2012).

Using the automated embryo placement systems (AEPS) previously described (Mandrell et al. 2012), 6 hpf dechorionated embryos were individually placed into a 96-well plate (1 embryo per well) prefilled with 90 µL of EM. Ten microliters of chemical from dilution plate 2 was added to each row of two 96-well plates. A final DMSO concentration was maintained throughout the experiment of 0.64 % (v/v). For each concentration, a total of 32 embryos were exposed. All exposure plates were sealed using parafilm to prevent evaporation and wrapped in aluminum foil to prevent light exposure. During this development period, zebrafish embryos are able to adapt to the dark and develop normally (Kokel et al. 2013). Exposed plates were stored in 28 °C incubator, and embryos were statically exposed until 120 hpf (5 dpf). At 24 hpf, embryos were assessed for PMR (as described below) and evaluated for mortality (MO24 = mortality at 24 hpf) (Truong et al. 2011). At 5 dpf, embryos were assessed for 17 morphological endpoints and collected in Zebrafish Acquisition and Analysis Program (ZAAP) (Truong et al. 2014).

Embryos were assessed for PMR using the custom-built Photomotor Response Analysis Tool (PRAT). The system uses a Prosilica GX3300 (Allied Vision, Stadtroda, Germany) with near infrared (NIR) band-pass filter to remove any influence of stimulus light. The lens is double telecentric (Navitar, Rochester, New York), mounted in an inverted manner beneath the plate holder to allow for imaging with minimum distortion and perspective interference. Imaging illumination is accomplished with a NIR (850 nm) Backlight (Smart Vision Lights, Muskegon, MI). PMR stimulus light is from two white L300 Linear Lights (Smart Vision Lights, Muskegon, MI). The system is controlled by custom hardware for timing the high-intensity light stimuli and system backlight. Video recording began immediately prior to light cycle initiation and captures 850 frames of digital video, recorded at 17 frames s-1. The light cycle consists of 30 s (sec) of background (prior to the first light pulse), a short pulse of light, 9 s before the next pulse, a second pulse of light, and then 10 s of dark.

To analyze the videos, a custom MATLAB program (Mathworks, Natick, MA) was used to compute a movement index for each frame stamp (the pixel differences between frames). The program output was processed using custom R scripts (R Core Team 2014) to discard the lag time that occurs between when the video recording was initiated and the light cycle begins/ends. A time stamp was created from the frame stamp by taking the first stamp after the recording started, setting that as 0 s, and then taking the floor of the sequential frame stamps.

### Quality control, identifying outliers, and quantifying expected responses

All subsequent analysis began with the processed data described above and was implemented using the R language (R Core Team 2014). First, we checked for aberrant background responses in individual plates and chemical sets by comparison with global patterns across all experiments. A total of 66 chemical sets (approximately 6 %) were rerun in full (both plates) after evaluation of the controls to identify those lacking post-light excitatory responses via either background (pre-light) peak movement index higher than the excitatory (post-light) peak, or no difference in movement from background to excitatory interval. After confirming lack of batch effects, these rerun data overwrote the original data in the results reported here.

Next, we removed wells annotated as having embryo mortality (MO24), as described in *Experimental Design*. For the analysis, the recorded periods were truncated to assure equivalence in recorded experimental period for all chemicals. Outlier detection was applied independently to each chemical concentration and time point combination. Movement values between the top 80–99 % quantiles (ignoring zero-movement values) were fit by a shifted gamma distribution using a maximum likelihood approach. An outlier was then defined as any movement value with a Bonferroni-corrected *p* value <0.05/*n*, where *n* is the number of movement values used to fit the model. This stringent cutoff criterion was chosen to remove outliers while preserving natural variation.

Additionally, we implemented a filter based upon departure from expected responses across all negative control (i.e., DMSO-only) wells. This filter prevents any significant calls from being made when the control fish are less responsive than expected. The peak-mean movement values within the excitatory interval were fit to a gamma distribution using a maximum likelihood approach, where a peak-mean movement value was defined as the maximum mean movement value of exposed fish for each chemical within that interval. Significance calls were prevented for any chemical with a peak-mean movement *p* value <0.05.

### Analysis of chemical-associated activity

Our analysis was designed to account for the unusual distributional properties of developmentally normal, spontaneous movement responses, and avoid bias toward one side of a bidirectional behavioral response where either direction could represent aberrant health effects of a chemical. Post-filtering (described in the previous section), the remaining experimental period (21–48 s) was subdivided according to light pulses: background (B) = 9 s prior to first pulse (21–29 s); pulse_1_ = first pulse of light; latency_1_ = 1 s immediately following first pulse; excitatory (E) = 8 s between light pulse (32–39 s); pulse_2_ = second light pulse; latency_2_ = 1 s immediately following second pulse; refractory (R) = 7 s after second pulse (42–48 s).

The statistical analysis of activity considered only the background (B), excitatory (E), and refractory (R) intervals. The overall pattern of activity within each B, E, or R interval was compared to that interval’s negative control (0 μM) activity using a combination of percent change (−50 % change from control for hypoactivity; 75 % change from control for hyperactivity) and a Kolmogorov–Smirnov test (Bonferroni-corrected *p* value threshold = 0.05/5 concentrations = 0.01). The percent change thresholds for hypo- and hyperactivity were parameterized so that the distributions of negative control responses were equivalent across activity-associated chemicals (i.e., “hits”). The Kolmogorov–Smirnov test compared the empirical cumulative distribution function (eCDF) between the chemical-treated samples and the negative control. For each chemical concentration set of *n* = 32 embryo wells, the treated wells were compared to vehicle (negative control) wells across two replicate plates for each chemical. This permits scalability of the procedure to custom batches of chemicals in either new experiments or retests (see “Quality control, identifying outliers and quantifying expected responses”).

The 5 dpf developmental morphology data, as described in Truong et al. (2014), were used to estimate the predictive value of this early, 24 hpf response. We modified the calculation of statistical significance from that paper in order to account for changing proportions from censored wells. Fisher’s exact test was used to define a morphology lowest effect level (LEL) if the incidence of the 5 dpf morphological endpoints were significantly different between the control fish and a concentration of chemical. Fish indicated as MO24 or 5 dpf mortality (MORT) were not included in the count data used in the test. The relative risk (RR) associating movement at 24 hpf (early effects) and the morphological endpoints measured at 5 dpf (later effects) was estimated from the number of chemicals positive for both early and late effects (true positive = TP); positive for early effects but negative for later effects (false positive = FP); negative for early effects but positive for later effects (false negative = FN); and negative for both early and late effects (true negative = TN), as:

$${\raise0.7ex\hbox{${\left( {\frac{\text{TP}}{{{\text{TP}} + {\text{FP}}}}} \right)}$} \!\mathord{\left/ {\vphantom {{\left( {\frac{\text{TP}}{{{\text{TP}} + {\text{FP}}}}} \right)} {\left( {\frac{\text{FN}}{{{\text{FN}} + {\text{TN}}}}} \right)}}}\right.\kern-0pt} \!\lower0.7ex\hbox{${\left( {\frac{\text{FN}}{{{\text{FN}} + {\text{TN}}}}} \right)}$}}.$$

## Results

**Fig. 1:**
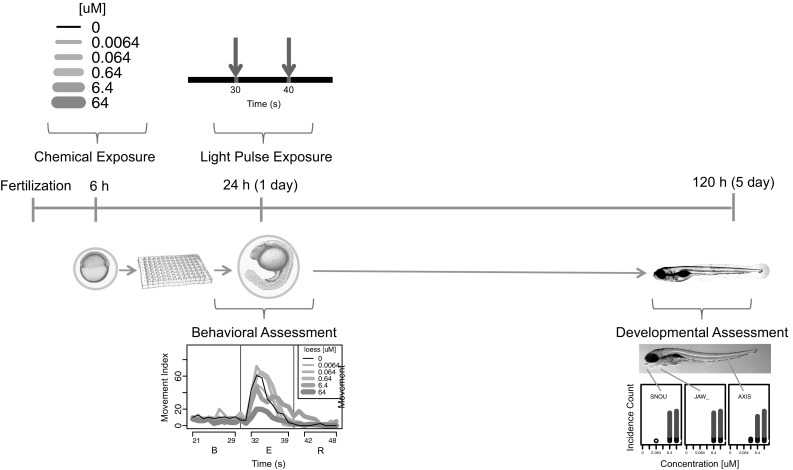
Experimental design. For each of 1060 chemicals, the chemical exposure at 6 hpf saw one of 5 concentrations of a given chemical added to individual embryo wells in 96-well plates. At 24 hpf, plates were subjected to light pulse exposure (at 30 s and 40 s) for the PMR assay. Movement was recorded across the short (<1 min) interval and then converted into the concentration–response profile shown under behavioral assessment. At 5 dpf, plates were assessed for specific developmental malformations, an example of which is shown under developmental assessment

**Fig. 2:**
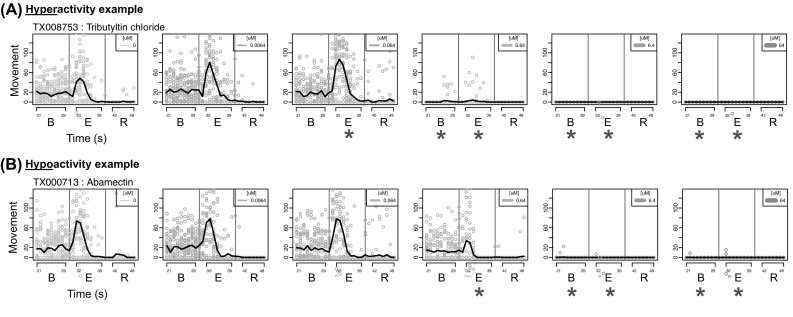
Detecting significant alterations in movement. The *top panel* shows movement (*vertical axis*, dimensionless index) versus time (*horizontal axis*, in sec) across all concentrations, increasing from *left* (control wells in the absence of chemical) to *right* (highest concentration = 64 µM). The *vertical red lines* indicate the timing of light pulses. Each circle represents movement at a given time for a replicate embryo well (32 replicates per chemical concentration combination). A locally weighted regression (LOESS) fit was plotted as a *solid black line*. The experimental intervals modeled in the behavioral analysis included background (B), excitatory (E), and refractory (R). *Red asterisks* denote significant responses for a particular concentration–time interval combination. The hyperactivity example chemical shown, tributyltin chloride, had an estimated lowest effect levels (LEL) of 0.064 μM for hyperactivity within the E interval and an LEL of 0.64 μM for hypoactivity within the B interval. The hypoactivity example chemical shown, abamectin, had estimated LELs of 6.4 μM in B and 0.64 μM in E (color figure online)

Data were collected from embryos exposed to each of 1060 ToxCast Phase-I and Phase-II chemicals (see “Chemicals”). The experimental design in Fig. 1 illustrates dechorionated embryonic zebrafish exposed to vehicle control plus five concentrations at 6 hpf, a photomotor response (PMR) assay at 24 hpf, and evaluation of 17 developmental malformation endpoints at 5 dpf. The response to the 24 hpf behavioral assay was interrogated, and movement profiles were established that characterized bidirectional responses to chemical exposure across a common five-point concentration range (see “Methods”). Representative hypo-/hyperactivity-associated chemical profiles are shown in Fig. 2. Full concentration response results for all chemicals are given in Supplemental Table 1.

### Global patterns of response and reliability

While individual sample (embryo) wells displayed variability in the magnitude of response, by considering the nonparametric distribution of all samples, a remarkably consistent pattern of activity emerged in control wells (see leftmost plots in Fig. 2). The background (B) interval was characterized by basal activity with moderate variance but absence of distinct activity peaks. The excitatory (E) interval displayed a single peak of hyperactivity with high variability as to the absolute peak movement and then immediately dropped to a movement level below that of B. The refractory (R) interval was characterized by a significant drop in movement compared to B, with inactivity lasting beyond the time interval measured.

The 9 sets of embedded chemical triplicate (each sourced separately) included in the data were used to assess reliability of activity calls. Using the same metric of assay concordance estimated for the ToxCast™ in vitro systems (http://epa.gov/ncct/toxcast/data.html), the reliability of activity calls was 89 %. For example, all three replicate sets for Allethrin exhibited the extremely rare refractory (R) hyperactivity response (observed in less than 1 % of all chemicals) at the same 64-μM concentration. Therefore, we concluded that the reliability was sufficient to collapse the embedded triplicate and report subsequent results with respect to the set of 1,060 unique substances.

**Fig. 3:**
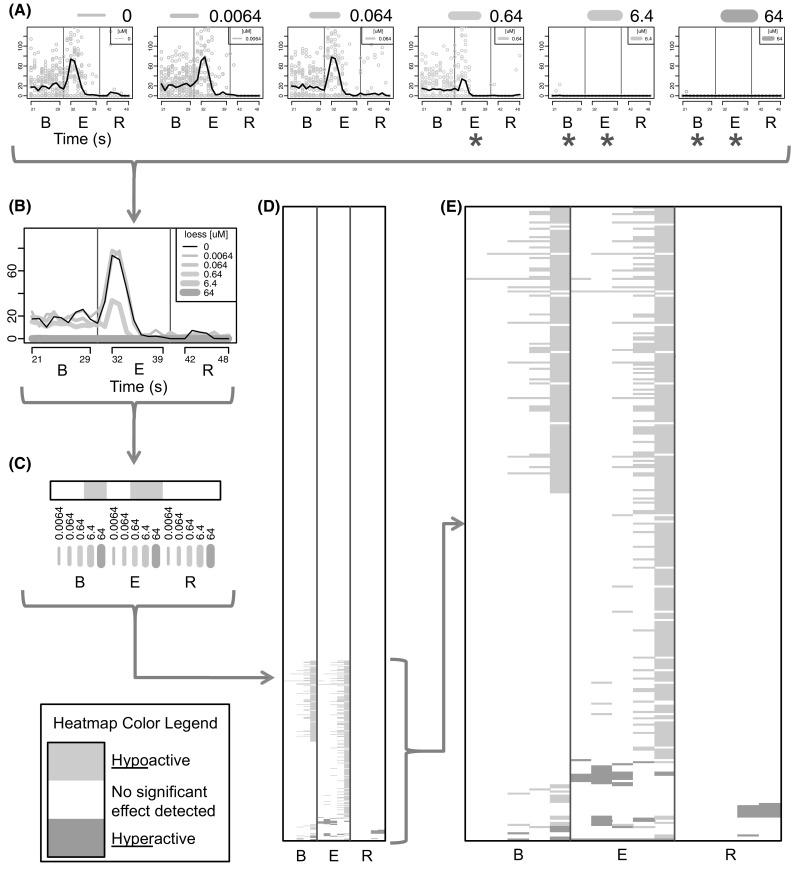
Response patterns for all chemicals across concentrations. In part A, movement versus time is plotted for all concentrations from *left* (control wells in the absence of chemical) to *right* (highest concentration) for a single chemical (abamectin, as in Fig. 2). *Red asterisks* denote significant responses for a particular concentration–time interval combination. In part B, the concentration–response data for the given chemical have been overlaid as a locally weighted regression (LOESS) fit, plotted with *line thickness* proportional to concentration of chemical. The LOESS fit for control embryos (wells in the absence of chemical) is plotted as a *solid black line*. In part C, the significant responses are collapsed into a heatmap vector (*row*) colored according to hyperactivity (*orange*) or hypoactivity (*blue*) at a given concentration and time interval. For the given chemical, only hypoactive significant responses were observed. In part D, the significant response vector for the given chemical is added as a single row in a heatmap for all chemicals (one chemical per row). The colorless portion of the heatmap represents the 764 chemicals without significant activity. In part E, the portion of the heatmap containing the 296 chemicals showing significant activity within at least one interval has been expanded (color figure online)

In total, 296 chemicals showed statistically significant activity in at least one interval (Supplemental Table 1 and Fig. 3). Of these significant chemicals, considering the lowest effect level (LEL) as the most potent (i.e., smallest concentration of chemical) response *within* a given time interval across all concentrations, there were 148 hypoactive and 5 hyperactive in B, 260 hypoactive and 18 hyperactive in E, and 7 hyperactive in R. The LEL overlap across intervals consisted of 12 out of 153 that were unique to B, 136 of 278 that were unique to E, and 6 of 7 that were unique to R. Beyond totals, considering the LEL as the most potent response *across* time intervals for a given chemical revealed the E interval as most sensitive in terms of detecting the most chemicals (171) at the lowest concentration. The B interval was the most sensitive for only 13 chemicals, and although rare, the 7 chemicals hyperactive within the R interval defined the LEL across intervals. These results indicate that each experimental period is differentially informative and that light-elicited behavioral changes in E tend to be most sensitive to chemical perturbation.

The LEL was chosen as the unit of analysis within each interval for several reasons illustrated by Fig. 3. The most sensitive effect (i.e., defining an LEL as the most potent chemical concentration associated with a significant effect) should be the most specific behavioral indicator. While hypoactive behavioral effects were observed for some chemicals at higher concentrations than those at which a hyperactive LEL was observed, *zero* chemicals across this diverse 1060 set caused significant reduction in movement followed by hyperactivity at a higher concentration. This point is extremely important when considering a behavioral endpoint that can take on three states (i.e., hyperactive, hypoactive, or insignificant change), because the dose–response may appear “non-monotonic” with respect to absolute movement. For example, a low-dose hyperactive response may trend toward hypoactivity at higher doses (e.g., tributyltin chloride or butylparaben), but the intermediate doses may be observed to pass through an apparent “no effect” response. This finding highlights the importance of a concentration–response design in assessing behavior, where a single concentration may happen to coincide with the point at which a response is neither significantly hyper- nor hypoactive as it transitions from significant hyperactivity at lower concentrations on the way to hypoactivity at higher concentrations. Moreover, because many of these high-concentration movement reductions were associated with mortality, it is likely that these effects were indicative of toxicity pathways leading to death.

Considering hypo- or hyperactivity LELs defined over the light intervals specified, we classified chemicals as eliciting light-*in*dependent movement alterations, light-dependent movement alterations (i.e., PMR specific), or indistinguishable from the observed data. The concentration–response profiles allowed us to infer light dependence for chemicals eliciting a post-light response (in E or R) at a concentration more potent than that at which significant (or absence of) B responses were observed. We found that 178 chemicals met this criterion of light-dependent movement alteration as the most sensitive effect (Supplemental Table 2). The LELs were distributed as 25 hyperactive and 153 hypoactive. The chemicals included several conazoles, thiocarbamates, metals, phthalates, toluenes, and the androgenic steroids 17-methyltestosterone, 17beta-trenbolone, 4-androstene-317-dione, and 5alpha-dihydrotestosterone.

In addition to evaluating signature profiles, we assessed the association of a chemical’s tendency to partition between hydrophobic and hydrophilic phases (LogP) with its ability to impact nervous system development in the PMR assay. Chemicals with higher LogP should be more permeable across biological membranes. However, as with a recent meta-analysis of zebrafish results across laboratories and experimental designs (Ducharme et al. 2013), we did not find significant evidence of LogP trending with effects across all chemicals in this behavioral assay. The absence of observed correlation may be due in part to the dechorionation step prior to chemical exposure, which facilitates uptake.

### Diverse modes of action are detectable by this assay

Examining chemicals having known bioactivity that displayed particular response patterns across intervals suggests that several modes of action are detectable by this assay, including direct binding to neurological elements, indirect signaling disruption, muscular contractile disregulation, and disruption of normal structural development. For example, the PMR assay was adept at detecting insecticides shown to target acetylcholinesterase, such as malathion (Cook et al. 2005) and all three forms of chlorpyrifos tested (Levin et al. 2003; Li et al. 2014; Yang et al. 2011). Moreover, this assay corrected identified the oxon metabolite of chlorpyrifos as more potent than the parent compound (6.4 vs. 64 μM, respectively). The thiocarbamate pesticides cycloate, dazomet, disulfiram, molinate, sodium dimethyldithiocarbamate, thiobencarb, thiram, and vernolate, known to cause notochord malformation resulting in neuronal and muscular disruptions, were identified by this assay but not associated with mortality (Haendel et al. 2004; Tilton et al. 2006; Tilton and Tanguay 2008). Not only does the PMR assay detect strictly nervous system or canonical neurotoxic modes of action, but it also identifies chemicals disrupting gross structural development, such as tributyltin chloride, tributyltin methacrylate, and triphenyltin hydroxide, which showed behavioral effects at concentrations lower than those at which morphological abnormalities were observed (McGinnis and Crivello 2011; Micael et al. 2007).

We also find several examples of chemical structural classes related to activity. None of the eight compounds from the thiophosphate alkyl subclass (tribufos, chlorethoxyfos, disulfoton, ethion, phorate, fosthiazate, terbufos, ethoprop) were active at 24 hpf. However, eighteen other thiophosphates, including fenitrothion, fenthion, methyl parathion, parathion (metabolized in vivo to Paraoxon), azinphos-methyl (gusathion), malathion, and methidathion, were all hypoactive in the post-light E interval, presumably via known acetylcholinesterase-related mechanisms. All five strobins included in this chemical set showed evidence of hypoactivity, with fluoxastrobin, trifloxystrobin, pyraclostrobin, and picoxystrobin having significant effects in the B and E intervals, and azoxystrobin’s otherwise significant hypoactivity censored by excess MO24 (mortality at 24 hpf). This may represent a mitochondrial adverse outcome pathway (AOP), where these fungicides act to inhibit the respiratory chain at the level of complex III (Bartlett et al. 2002). Indeed, beyond these strobins, other chemicals with known mitochondrial targets, such as rotenone (Ai et al. 2014; Navarro et al. 2010), pyridaben (Navarro et al. 2010), and fenpyroximate (Z,E) (Shiraishi et al. 2012) displayed hypoactive responses.

We found that significant movement within the refractory interval was very rare, with only seven chemicals showing hyperactivity: hexaconazole, carbofuran, cypermethrin, methomyl, prallethrin, S-bioallethrin, and allethrin. The response patterns suggested delayed recovery from light-stimulated excitation or sustained hyperactivity with disruption of the stereotypical photomotor response. These chemicals include 4 pyrethroids known to affect ion channels, a known neurotoxic triazole organophosphate (hexaconazole), and two carbamates (methomyl and carbofuran, which was once prescribed as an anxiety treatment in humans) known to affect acetylcholinesterase activity (Ensibi et al. 2014).

The most potent hypoactive chemicals at 24 hpf (LEL ≤ 0.064 μL) were emamectin benzoate (Carmichael et al. 2013), rotenone (Swarnkar et al. 2011), triphenyltin hydroxide (Zsombok et al. 1997), thiram (Lee and Peters 1976), mercuric chloride (Yasutake et al. 2010), dapsone (Waldinger et al. 1984), and fluconazole (van Schie et al. 2011). Although all of these compounds have been implicated in nervous system perturbations related to neuromuscular dysfunction, this behavioral assay detected diverse mechanisms of action. For example, rotenone is a pesticide that promotes Parkinson-like behavior upon exposure by inhibiting mitochondrial complex I (Bretaud et al. 2004), while emamectin benzoate interferes with γ-aminobutyric acid (GABA)-gated ion channels in the nervous system (Carmichael et al. 2013).

### External data integration to identify specific molecular targets

To investigate whether chemicals associated with neurobehavioral phenotypes in our data were associated with biological pathways that manifest in neuroactive responses, we performed a statistical enrichment analysis of our zebrafish 24 hpf behavioral (in vivo) data in conjunction with biochemical assay (in vitro) results from (Sipes et al. 2013), where the ToxCast Phase-I and Phase-II chemicals were screened across 331 cell-free enzymatic and ligand-binding assay targets. The analysis considered statistical enrichment (Fisher’s exact test *p* < 0.05 and odds ratios >1) of chemical “hits” in the 24 hpf behavioral results associated with individual in vitro assays (e.g., Novascreen human peripheral-type benzodiazepine receptor-binding assay). We also performed the same enrichment analysis in association with high-level assay groupings from Table 1 in Sipes et al. which aggregated assays into functional groupings such as aminergic G-protein coupled receptor binding (32 assays), ion channel binding (7 assays), or cholinesterase enzyme inhibition (3 assays).

Considering the full concentration–response profile to define light-dependent effects (see Supplemental Table 2 as described earlier), chemicals eliciting light-dependent LELs were associated with hits on assay groups of nuclear receptors, transporters, CYPs, ion channels, and aminergic GPCRs. The individual assays associated with light-dependent LELs were the binding assays probing VMAT2 (vesicular monoamine transporter), PBR (peripheral benzodiazepine receptor or TSPO), DAT (dopamine transporter), NET (norepinephrine transporter), PXR (pregnane-X receptor), PR (progesterone receptor), AR (androgen receptor), and CAR (constitutive androstane receptor), as well as enzymatic inhibition assays for MAOB (monoamine oxidase B) and several CYPs (human CYP19A1, CYP1A1, CYP2B6, CYP1A2, CYP2D6). This behavioral assay identified light-dependent hypoactivity for 15 of the 16 chemicals that were hits in the external, in vitro MAOB assay.

We also considered more general response prototypes of hyper- or hypoactivity within each experimental light interval as chemical subsets for mapping against the targeted, in vitro results. These response prototypes were defined according to LELs within intervals. For example, 123 chemicals displayed the (B,E,R) prototype = (−1, −1, 0), corresponding to hypoactivity LELs within both the background and excitatory intervals. We found that this prototype indicating general hypoactivity was associated (*p* < 0.05) with many of the same assays as the light-dependent chemicals, plus assays for 5-HT (serotonin receptor) and several opioid receptors. Thus, chemicals eliciting abnormal behavioral responses at 24 hpf in zebrafish are enriched for activity within in vitro assays probing genes that have known roles in canonical neurological signaling. Variation in these genes, such as monoamine oxidase A (MAOA), has been associated with a variety of psychiatric traits and sensitivity to chemical therapeutics (Ziermans et al. 2012).

On the flip side, statistically significant in vitro assay associations with negative chemicals, i.e., those with prototype = (0, 0, 0), had “protective” odds ratios (Fisher’s exact test *p* < 0.05 and odds ratios <1). In this context, the interpretation of a significant odds ratio <1 is that the compounds inactive in this behavioral assay were less likely to “hit” the given targeted assays. The in vitro assay groups fitting this criterion included cholinesterases, nuclear receptors, transporters, CYPs, ion channels, and aminergic GPCRs. Thus, chemicals that were negative in this 24 hpf behavioral assay displayed essentially the inverse relationship with in vitro assays targeting plausible neuroactivity pathways. Importantly, there were no significant, positive odds ratios that associated the (0, 0, 0) behavioral prototype with any individual assay or assay group from Sipes et al.

### Predictive power of 24 hpf behavioral results

**Fig. 4:**
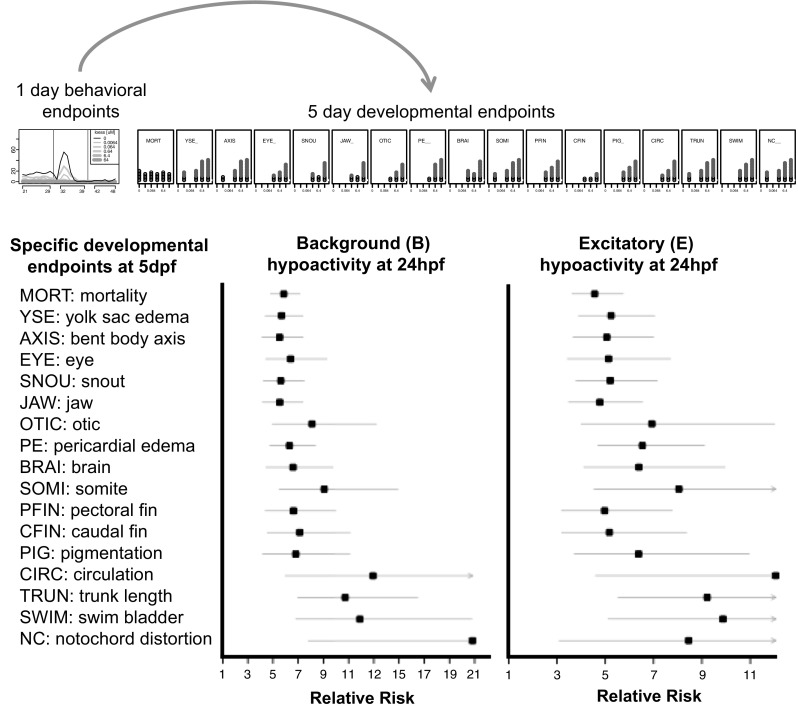
Predictive power of 24 hpf behavioral data. For chemicals eliciting significant hypoactivity within the B or E time interval in the 24 hpf behavioral data, the relative risk (RR) was estimated for each 5 dpf developmental endpoint. The plots show the estimated RR (*solid square*) and 95 % confidence intervals. For each plot, the *vertical line* indicates a RR = 1 (i.e., no evidence of association). The 24 hpf behavioral and 5 dpf developmental profiles atop the plots are for the chemical thiram

Taking advantage of the multi-scale nature of the phenotypic measurements collected, we integrated across 24 hpf behavior and follow-up 5 dpf development endpoint to understand relationships amongst endpoint types. Figure 4 shows the prospective association relative risk (RR) between hypoactive movement at 24 hpf and the morphological endpoints measured at 5 dpf. We found that these early behavioral responses were predictive (RR *p* value <0.05) of 17 specific developmental abnormalities—including 18 of 22 chemicals causing notochord defects—and mortality measured at 5 dpf. Moreover, many 24 hpf behavioral LELs were more potent than those for gross morphological effects, such as the hypoactive LELs for all three neurotoxicant avermectins tested: abamectin, milbemectin, and emamectin benzoate (Lumaret et al. 2012). Additional value added from this assay is exemplified by responses such as that of thiram (a member of the ethylene bisdithiocarbamate class and the most potent 24 hpf hypoactive chemical), which was not associated with mortality at either 24 hpf or 5 dpf, though specific developmental defects (including notochord malformation) were observed at 5 dpf (see Fig. 4).

While confidence bands for the lower-incidence endpoints in Fig. 4 are wider than those for more common 5 dpf endpoints, all endpoint-wise RR for chemicals eliciting hypoactive responses in B or E is statistically significant in the positive direction (i.e., aberrant behavior at 24 hpf is associated with higher risk of developmental abnormalities at 5 dpf). Furthermore, the positive predictive values (PPV) of hypoactivity results for any 5 dpf endpoint, defined as the fraction of chemicals causing 24 hpf hypoactivity that caused at least one specific developmental abnormality at 5 dpf, were 90, 66, and 71 % for the B, E, and R intervals, respectively.

## Discussion

As an integrative measure of normal development, significant alterations in movement highlight chemicals representing several modes of action. Evaluating the photomotor response of chemical-exposed embryos can provide a holistic sensor, unachievable through focused cell-based systems or singular morphological phenotypic assessments. This is of significant value in a screening context employing a comprehensive suite of endpoints, where certain chemical-elicited responses may not manifest in strictly morphological changes observed at any single time point. Even without a specific mode of action linked to each 24 hpf behavioral positive chemical, aberrant behavior in an otherwise stereotypical response should be cause for additional scrutiny and may eventually refine existing AOPs or elucidate entire new pathways.

These results demonstrate that chemicals altering behavioral responses at an early time point (24 hpf) can manifest in later (5 dpf) developmental abnormalities, indicating that rapid behavioral screening can be a useful diagnostic. Moreover, chemicals associated with severe behavioral abnormalities may lead to developmental effects that are, as yet, unmeasured. In these data, we found 102 chemicals associated with 24 hpf behavioral endpoints yet *no* specific 5 dpf endpoint associations (Supplemental Table 3). These chemicals, when encountered at a critical, early exposure period, may alter the timing of the coordinated cascade of developmental events that normally lead to healthy adults. Compared to effects detected by manual, visual inspection at 24 hpf, we found that the majority of chemicals inducing aberrant behavior were either undetected or not identified until higher concentrations. This demonstrates added value of the 24 hpf behavior assay in a screening paradigm that includes morphological screens as in Truong et al. (2014) or end-stage scoring approaches as in Padilla et al. (2012). In addition to expanding the net for detection of adverse outcomes, combining this early, nondestructive assay with later endpoints should reduce false negatives. Given such a large, diverse chemical set, false negatives within a particular assay can arise for any reason, ranging from technical handling of chemicals preferentially affecting certain endpoints (especially if measured at different time points) to sampling variability within outbred populations. Therefore, the combination of evaluating both early behavioral endpoint and phenotypic responses in the same animals provides the holistic perspective necessary to warrant further study of active chemicals.

While measures have been taken to avoid false positives, the analysis here is tuned toward detection, since the goal was to screen a large set of environmental chemicals in full concentration–response format. This goal diverges from that of compound-similarity clustering approaches such as Kokel et al. (2010), because each chemical in our set of compounds is (or has been) in commerce and the environment. For this reason, they must be characterized to determine whether significant evidence of hazard exists across a broad concentration range. While our analysis took advantage of the magnitude of all data to define expectations and identify outliers, the statistical framework described here can accommodate smaller-scale experiments, because activity thresholds are ratio-based comparisons to local controls, rather than requiring an absolute unit change in spontaneous movement.

The advantages of the statistical framework developed here over more traditional alternatives, such as paired second-to-second tests, include: (1) ability to detect changes in both centrality (i.e., average movement) and distributional shape; (2) robustness in the face of changing, nonstandard distributions in a measurement of a spontaneous phenotype; and (3) consistency, in that explicit perturbations (i.e., light pulses) define more stable responses than periods of only a few seconds within an experimental interval. While the time periods could be subdivided into smaller units of analysis for fine-scale modeling of particular responses, we found that the asynchrony between individual embryo wells on a second-to-second time scale overwhelms treatment-associated variability—especially if more than the samples comprising the interquartile range are analyzed as in the pooled embryo method of (Kokel et al. 2010). However, when considered across a multi-second experimental interval, consistencies arise, and by using all samples to characterize the effects of a given chemical, we capture a more representative sample of the full variability present in the population. Indeed, our analysis shows high reliability amongst separately sourced, blinded replicate sets.

Importantly, with such a noninvasive, automated endpoint, perturbations of AOP-associated molecular targets could be assessed through morpholino knockdown and/or RNA rescue (Andreasen et al. 2006; Franzosa et al. 2013; Miller et al. 2014; Reimers et al. 2006; Tilton et al. 2006; Tilton and Tanguay 2008). The scale of the results presented here, using an experimental design including high numbers of biological well-replicates, permits powerful enrichment analysis to confirm such molecular targets. We have presented integrated analyses spanning endpoints via follow-up within our experiment (24 hpf behavior to 5 dpf morphology) and external experiments (biochemical assays from Sipes et al.). This demonstrates that these results can augment the value of systematic data generated by ongoing efforts to realize the promise of modernized, sustainable approaches to environmental health protection as envisaged by the US National Research Council’s *Toxicity testing in the 21st century: a vision and a strategy* (2007). Accordingly, we present these rapid throughput, vertebrate screening results as an important resource for cross-species hazard assessment and encourage the use of these data for additional meta- and cross-platform analyses.

In conclusion, this rapid neurobehavioral assay identified chemicals displaying light-dependent and light-independent effects at an early developmental stage (24 hpf), predicted increased risk of later (5 dpf) developmental hazard, associated behavioral responses with in vitro endpoints targeting specific biological pathways, characterized a broad concentration–response profile for each chemical, and detected chemicals covering several potential modes of action. The experiments described here address statistical uncertainty concerns commonly associated with in vivo methods—such as low sample size and inadequate concentration–response—by engineering an efficient, rapid throughput system where these important design factors can be efficiently optimized. This system represents an efficient experimental platform for conducting neurobehavioral assessment early in vertebrate development that, due to its noninvasive nature, can be recombined with other experimental interventions and/or later outcome measures.

## Electronic supplementary material

Below is the link to the electronic supplementary material.

Supplementary material 1 (CSV 67 kb)

Supplementary material 2 (CSV 11 kb)

Supplementary material 3 (CSV 6 kb)
